# Effectiveness of PBL methodology in a hybrid dentistry program to enhance students’ knowledge and confidence. (a pilot study)

**DOI:** 10.1186/s12909-018-1392-y

**Published:** 2018-11-20

**Authors:** Ebtissam M. Al-Madi, Sree Lalita Celur, Mamoona Nasim

**Affiliations:** 1 Department of Restorative Dental Sciences, College of Dentistry, King Saud University, Riyadh, Kingdom of Saudi Arabia; 2 College of Dentistry, Princess Nourah bint Abdulrahman University, Eastern Ring Road, P.O. Box: 84428, Riyadh, 11671 Kingdom of Saudi Arabia; 3 Department of Anatomy, Histology & Embryology, University of Health Sciences, Lahore, Pakistan

**Keywords:** Dental education, Problem-based learning, Self-reported confidence, Teaching methods, Knowledge retention, Confidence sustainability

## Abstract

**Background:**

Knowledge and self-confidence are two critical determinants of future success of dental students. The present pilot study was conducted with an objective to simultaneously assess both knowledge and confidence gained by dental undergraduate students in the Head and Neck Anatomy course by employing didactic lecture-based and problem-based learning methods.

**Methods:**

A paper-based assessment tool comprising of 30 Multiple choice questions to assess knowledge, followed by a Likert’s scale to assess students’ confidence to answer the given knowledge question was designed. This tool was used in a cohort of first year dental students before the commencement of Head and Neck Anatomy course (Pre-course), immediately after the completion of Head and Neck Anatomy course (Post-course), and again in third year before the same cohort entered their clinical courses (Pre-clinics). The difference in students’ knowledge and confidence through both pedagogies was evaluated by Paired ‘t’ test. Pearson correlation analysis was done to determine the correlation between knowledge scores and self-reported confidence.

**Results:**

A statistically significant increase (*p* < 0.05) was noted in the mean knowledge and confidence scores in the post-course evaluation, through both didactic lecture-based and problem-based learning methods. On the other hand, a significant decrease (*p* < 0.05) in the mean knowledge and confidence scores of didactic lecture-based items in comparison to problem-based items was noted in the pre-clinics evaluation.. The post-course evaluation results yielded a Pearson correlation coefficient of *r* = 0.514, *p* = 0.002 for lecture-based items and *r* = 0.495, *p* = 0.003 for problem-based items, denoting a positive moderate correlation between the knowledge and confidence scores for both lecture-based and problem-based methods.

**Conclusion:**

A significant improvement in both knowledge and self-reported confidence demonstrated at the end of Head and Neck Anatomy course proves both didactic lectures and problem-based learning methods to be equally effective in a hybrid dentistry program in the short term. However, the non-significant reduction in the pre-clinics knowledge and confidence scores among the PBL lessons proves it to be a potent learning tool for long term retention of knowledge, and sustainability of confidence.

## Background

Having a comprehensible judgement of one’s own abilities and strengths enables a person to be self-confident [1]. This self-confidence in turn is an outcome of cognitive processing and transformation of various sources of information [2]. Although self-confidence is one of the most important generic competencies in academia [3], confidence without adequate background knowledge is potentially harmful [4]. Dental students must be self-confident of their knowledge to make the appropriate decisions for their patients [5, 6]. Poor alignment of knowledge and confidence results in either under confident or overconfident practitioners posing a threat to patients [7, 8].

Active learning strategies entice students into a congruent discussion and habit of critically analyzing situations, thus maximizing the impact of the material on the learners [9]. Problem Based Learning (PBL) methodology utilizes real problems as triggers and creates a learning environment that motivates students to get involved actively and think critically [10]. The knowledge thus gained would be retained and applied for problem solving in their future clinical practice [3]. This self-learning plays a vital role in boosting the confidence of students.

Several published studies evaluated the outcomes of problem-based and traditional methods at the curriculum and single course level with varying results [11–13]. However, in terms of test scores there were no significant differences in the short-term results in majority of studies [11]. Heijne-Penninga M found that students of student-centered PBL curricula showed a substantially better long-term knowledge retention than teacher-centered traditional curriculum students [14]. Similar results were obtained by Beers GW and Bowden S at a single setting [15].

The kinship of confidence and knowledge is known to accelerate the learning process, thereby improving student performance and competence. Literature search reveals few studies showing active learning techniques to enhance knowledge and confidence of health profession students [7, 16, 17], but no studies were found evaluating the effectiveness of PBL methodology to enhance both knowledge and confidence, specifically in a dentistry setting either at the curriculum or a single course level. In order to address this lacuna, a pilot study was conducted at a single course level of Head and Neck Anatomy in the College of Dentistry, Princess Nourah bint Abdulrahman University (CD-PNU). The CD-PNU adopted a hybrid dental program incorporating didactic lectures and PBL tutorials. This program spans for a period of 6 years, with the students learning the foundation subjects and undergoing pre-clinical training in the first 2 years after which they start clinical training on real patients from third year. Knowledge and understanding of head and neck anatomy and its applied aspects is fundamental for dental graduates, not only to perform oral surgical procedures but also to minimize associated complications. The Head and Neck Anatomy course, which is a 12-week block course for the first-year students at CD-PNU, is designed in such a way that factual knowledge is transferred through didactic lectures, while the topics requiring scientific inquiry are covered through 7-jump PBL methodology [18], so as to foster critical thinking and scientific inquiry.

Hence, the present study was conducted with a hypothesis that PBL has a positive effect on knowledge retention and self-confidence of dental students. The objectives of the study were 1) to evaluate the students’ knowledge and self-reported confidence before and after the completion of Head and Neck Anatomy course in the first year, by stratifying the data based on didactic lectures and PBL sessions; 2) to evaluate the retention of knowledge gained in Head and Neck Anatomy course and associated self-reported confidence just before entering the clinics in third year; and 3) to determine the correlation between knowledge scores and self-reported confidence.

## Methods

**Fig. 1:**
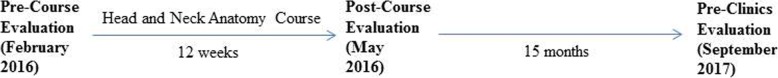
Time frame of the study

This cross-sectional pilot study was reviewed and approved by the PNU Ethical Review Board (IRB number: 2015/CDS/E/002) and was conducted at CD-PNU during the academic years 2015–16 and 2017–18 (Fig. 1). Since the study instrument was only in the form of a written assessment, the PNU Ethical Review Board approved that a verbal consent from the participants is adequate. The students were ensured that either participation or non-participation in the study would not influence their academic grades. Thirty-three out of 42 female students enrolled for the Head and Neck Anatomy course in the academic year 2015–16, voluntarily participated in the study. To assess the students’ knowledge, a paper based assessment tool comprising of 30 multiple choice questions (MCQs) of the single best type was designed and checked for construct validity. The MCQs were mainly of C2 or C3 level of Bloom’s taxonomy, as they were appropriate to examine the comprehension and application abilities of students. Out of these 30 MCQs, 15 were structured from the topics covered through didactic lectures, and the remaining 15 from the topics covered through PBL (Table 1). The MCQs to assess knowledge had 3 distractors and a correct answer and each item was scored as either 0 (incorrect) or 1 (correct). Each MCQ was followed by a confidence item on a 5-point Likert scale (0 = not sure at all, 1 = very unsure, 2 = somewhat sure, 3 = very sure, and 4 = extremely sure) to assess students’ self-reported confidence about the correctness and certainty of the answer. Fifteen MCQs based on didactic lectures could yield a maximum score of 15, and like-wise 15 MCQs based on PBL could yield a maximum score of 15. A maximum score of 60 could be yielded from the 15 confidence items based on didactic lectures, and similarly a maximum score of 60 could be yielded from the 15 confidence items based on PBL.

**Table 1:** Topics of Head & Neck anatomy course used for structuring MCQs

Didactic lectures	Problem based learning
Scalp, cranial cavity and Brain	Parotid gland
Temporal fossa	Infratemporal fossa
Oral cavity	Nasal cavity
Pterygopalatine fossa	Facial nerve
Triangles of neck	Trigeminal nerve

The same assessment instrument was used for pre-course, post-course, and pre-clinics evaluation. The pre-course evaluation was to have a baseline data to compare and correlate the post-course knowledge and self-reported confidence. The pre-clinics evaluation was done fifteen months after the delivery of the head and neck anatomy course, to determine the retention of knowledge gained by each instructional method.

Composite knowledge and confidence scores were calculated for each student by numerically summing the score for each MCQ and confidence item respectively. Statistical analysis was done using SPSS (IBM SPSS Statistics for Windows, Version 21.0. Armonk, NY: IBM Corp.). Mean and standard deviation of composite knowledge and confidence scores for the pre-course, post-course, and pre-clinics evaluation were calculated for the entire cohort, and the data followed the normal distribution. The difference in pre and post-course evaluation scores for the didactic lectures and PBL was evaluated by paired ‘t’ test, and the difference in post-course and pre-clinics evaluation scores for both the instructional methods was also evaluated by paired ‘t’ test. Pearson correlation analysis was done to determine the relation between knowledge scores and self-reported confidence of both didactic lectures and PBL.

## Results

### Changes between pre- and post-course evaluation scores

The mean knowledge score (lecture based) increased from 3.45 ± 1.73 to 6.76 ± 2.36 with statistical significance (*p* < 0.001), and the mean knowledge score (problem based) increased from 4.42 ± 1.66 to 5.97 ± 1.99 with statistical significance (*p* = 0.001) (Table 2). The mean confidence score (lecture based) increased from 18.06 ± 7.84 to 33.97 ± 10.17, and the mean confidence score (problem based) increased from 11.15 ± 8.86 to 29.42 ± 10.4 with a statistical significance (*p* < 0.001) (Table 3). No statistically significant difference was noted in the post-course evaluation knowledge scores of both the problem based and lecture based items (*p* = 0.14) (Table 2). However, a statistically significant improvement was noted in the confidence scores of the post-course evaluation with a higher mean score for lecture based items in comparison to problem based items. (*p* = 0.002) (Table 3).

**Table 2:** Changes in pre-course, post-course, and pre-clinics knowledge scores

Evaluation	Mean	Std. Deviation	Mean Difference	95% Confidence interval of the difference		‘*t*’ value	‘*p*’ value
				Lower	Upper		
Pre-course (lecture based)	3.45	1.73	−.97	−1.9	−.03	−2.1	.04
Pre-course (problem based)	4.42	1.66					
Pre-course (lecture based)	3.45	1.73	−3.3	−4.28	−2.32	−6.86	< 0.001
Post-course (lecture based)	6.76	2.36					
Pre-course (problem based)	4.42	1.66	−1.54	−2.39	−.69	−3.7	.001
Post-course (problem based)	5.97	1.99					
Post-course (lecture based)	6.76	2.36	.79	−.27	1.84	1.52	.14
Post-course (problem based)	5.97	1.99					
Pre-clinics (lecture based)	5.09	1.96	−1.67	−2.79	− 0.54	−3.02	.005
Post-course (lecture based)	6.76	2.36					
Pre-clinics (problem based)	5.9	2.06	−.07	−1.06	0.94	−0.12	.903
Post-course (problem based)	5.97	1.99					
Pre-clinics (lecture based)	5.09	1.96	−.81	−1.83	.19	−1.64	.11
Pre-clinics (problem based)	5.9	2.06

**Table 3:** Changes in pre-course, post-course, and pre-clinics confidence scores

Evaluation	Mean	Std. Deviation	Mean Difference	95% Confidence interval of the difference		‘*t*’ value	‘*p*’ value
				Lower	Upper		
Pre-course (lecture based)	18.06	7.84	6.9	5.24	8.58	8.43	< 0.001
Pre-course (problem based)	11.15	8.86					
Pre-course (lecture based)	18.06	7.84	−15.9	−19.87	−11.94	−8.18	< 0.001
Post-course (lecture based)	33.97	10.17					
Pre-course (problem based)	11.15	8.86	−18.27	−21.95	−14.59	− 10.13	< 0.001
Post-course (problem based)	29.42	10.4					
Post-course (lecture based)	33.97	10.17	4.54	1.84	7.25	3.42	.002
Post-course (problem based)	29.42	10.4					
Pre-clinics (lecture based)	25.24	8.46	−8.73	−12.99	−4.47	−4.17	< 0.001
Post-course (lecture based)	33.97	10.17					
Pre-clinics (problem based)	25.39	9.7	−4.03	−8.36	0.29	−1.89	.06
Post-course (problem based)	29.42	10.4					
Pre-clinics (lecture based)	25.24	8.46	−.15	−1.74	1.44	−.19	.85
Pre-clinics (problem based)	25.39	9.7					

### Changes between post-course and pre-clinics evaluation scores

The mean knowledge score (lecture based) decreased from 6.76 ± 2.36 to 5.09 ± 1.96 with statistical significance (*p* = 0.005), and the mean knowledge score (problem based) marginally decreased from 5.97 ± 1.99 to 5.9 ± 2.06 with no statistical significance (*p* = 0.903) (see Table 2). The mean confidence score (lecture based) decreased from 33.97 ± 10.17 to 25.24 ± 8.46 with a statistical significance (*p* < 0.001), and the mean confidence score (problem based) decreased from 29.42 ± 10.4 to 25.39 ± 9.7 with no statistical significance (*p* = 0.06) (Table 3). No statistically significant difference was noted in the pre-clinics evaluation knowledge and confidence scores of both the groups (Tables 2 & 3).

### Correlation among knowledge and confidence scores

**Fig. 2:**
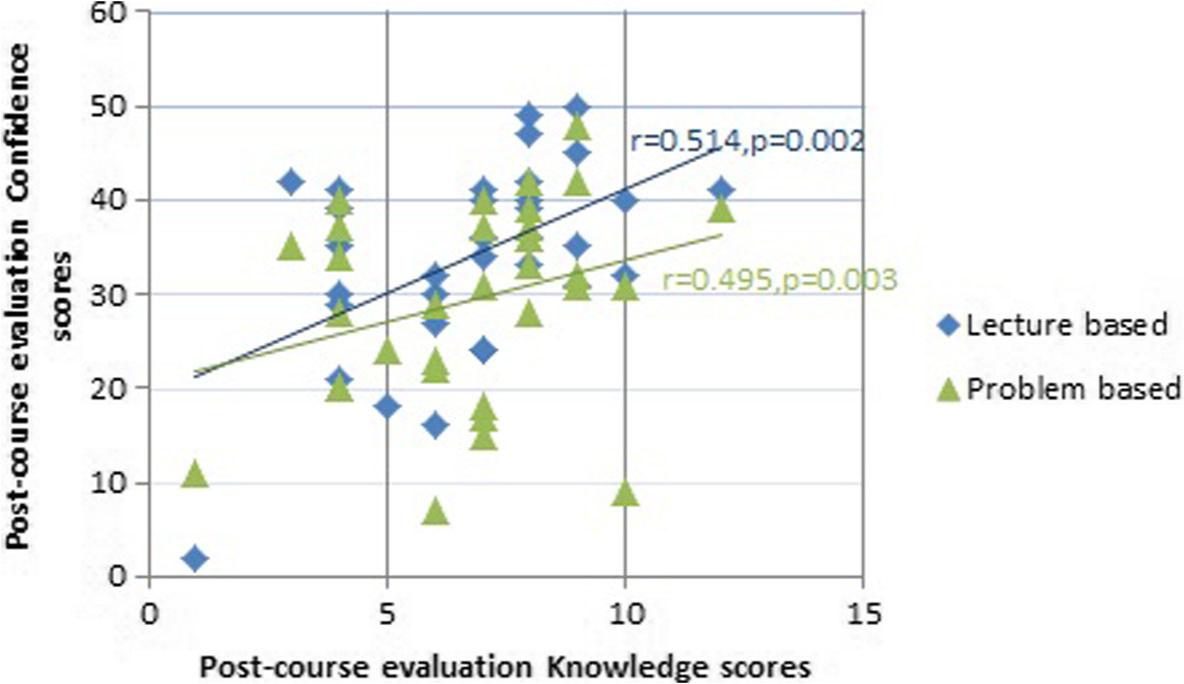
Correlation of Post-course knowledge and confidence scores

There was no statistically significant correlation among the knowledge and confidence scores in the pre-course and pre-clinics evaluation. The post-course evaluation results yielded a Pearson correlation coefficient of *r* = 0.514, *p* = 0.002 for the lecture based items and *r* = 0.495, *p* = 0.003 for the problem based items (Fig. 2). Although the obtained ‘*p*’ value is statistically significant, the obtained ‘*r*’ value denotes a positive moderate correlation between the knowledge and confidence scores for both lecture based and problem based methods.

## Discussion

PBL is encouraged by CD-PNU as it encompasses active learning with preset learning objectives [19]. Real life clinical scenarios are used as triggers to motivate learners to take responsibility for their own learning, which ultimately improves their self-confidence and abilities in practically dealing with those situations in future [20]. The Head and Neck Anatomy course was designed to incorporate both the teacher centered and student-centered pedagogies. Students showed significant improvement in both knowledge and confidence by both the pedagogies upon completion of the course. This is analogous to the report of Kang KA et al. showing significant improvement in the knowledge, confidence and satisfaction with all the three educational modalities of lecture based, problem based, and simulation with PBL, in nursing students [21].

No statistically significant difference in the post-course knowledge scores of both didactic lectures and PBL portray that both the learning methodologies are at par, and endorse the conclusion of Bassir SH, Sadr-Eshkevari P, Amirikhorheh S, and Karimbux NY that PBL does not negatively influence the acquisition of factual knowledge in dental students [12]. However, other literary evidence showed that PBL had a positive effect, with students gaining higher theoretical scores compared to traditional lecture based teaching which is not similar to our findings [13, 22]. This contrast could be attributed to the local academic setting, where students have no formal education of this type in Higher secondary school, and they are introduced to the concepts of PBL for the first time at their undergraduate level of professional study. The other factors which could be responsible for this contrast are the differences in study design and outcome variables [12].

Although the students’ performance improved after the course by both didactic lectures and PBL, the assessment of knowledge scores in the pre-clinics evaluation showed that there was a significant decrease in the students’ knowledge gained by didactic lectures, which was not the case with PBL. Thus, PBL helped in both short term and long-term retention of knowledge similar to the findings of Beers GW and Bowden S in nursing students [14]; and Heijne-Penninga M et al. in medical students [15]. These long term gains achieved by PBL is beneficial for the incorporation of basic sciences knowledge into clinical practice. In contrast to PBL, team based learning (TBL) led to large gains in knowledge only over the short term [23].

Unlike knowledge scores, confidence scores in the post-course evaluation were on a higher range for the lecture based items in comparison to the problem based items. This partial lack of congruency between knowledge gained and self-reported confidence is primarily because of inadequate sensitization to the process of self-directed learning and self-assessment as the students selected for the study are still in the first year of dental school. Secondly, the self-reported confidence scores would have been more correlative with the knowledge scores, when the students are driven to apply the gained knowledge in practically solving problems and treating patients in a clinical setting, which provides a more accurate means of self-evaluation. This assumption is based on the results of Callis et al., which displayed that students of hybrid-PBL curriculum were better at applying basic science knowledge to a clinical case in comparison to students of traditional lecture based curriculum [24].

A moderate positive correlation of knowledge and self-reported confidence through both the pedagogies, was demonstrated in the post-course evaluation of the current study. This is in contrast to Tousignant & DesMarchais findings, in which they reported a very weak correlation of student self-assessment and performance in a problem-based learning medical program, attributed partly to the stress associated with summative assessment [6]. This optimum intellectual confidence is essential for the creation of a non-hazardous environment of clinical practice, and is considered a positive sign in providing quality care to the patients [25]. This positive correlation of knowledge and self-reported confidence dissolved by the time of pre-clinics evaluation, which might be because the students still did not have any opportunity to integrate their knowledge with practice. It is also presumed that the positive correlation of knowledge and confidence would evolve back, as the transition of the students’ theoretical knowledge progresses into clinical practice, by which time the students would be able to identify their own strengths and weaknesses adequately [26].

As the confidence scores were self-reported in this study, inter-rater variability might have influenced the results. Moreover, as there was no incentive given to the students for taking the pre-course, post-course, and pre-clinics evaluation, some students may not have answered the knowledge questions to the best of their ability. Other methodological limitations to this study include using a single course setting restricted to female students, and using only MCQs to assess student knowledge. Addressing these issues, further continuation of the study is suggested, to determine the application of basic science knowledge gained through PBL in solving the problems encountered in clinical practice. The current study also gives scope for long-term studies evaluating the retention of knowledge acquired by both lecture-based and PBL methods. Similar studies done throughout the curriculum would provide a true reflection of the effectiveness of PBL methodology in a hybrid dentistry program.

## Conclusion

A significant improvement in both knowledge and confidence was demonstrated at the end of Head and Neck Anatomy course delivered for the first year dental students by both didactic lectures and PBL methods. This improvement in both the parameters at the conclusion of the course indicates both student centered and teacher centered learning methods to be equally effective in a hybrid dentistry program. The non-significant reduction in the pre-clinics knowledge and confidence scores resulting from PBL proves it to be a potent learning tool for long term retention of knowledge, and sustainability of confidence.

## Abbreviations

CD-PNU: College of Dentistry, Princess Nourah bint Abdulrahman University
MCQs: Multiple choice questions
PBL: Problem based learning
PNU: Princess Nourah University
